# A Cumulative Action of Alcohol

**Published:** 1896-11

**Authors:** 


"A Cumulative Action of Alcohol."—Alcohol, like other substances of a narcotic nature, has the power, when taken frequently, even in small quantities, to create a diseased appetite for more, which may become uncontrolable, and its gratification destructive."

The above statement, signed by thirty-six physicians of the United States and Canada, more or less distinguished in connection with the subject, appears in the "*School Physiology Jonrnal*," "a magazine published to help teachers in teaching the Physiological Temperance, which is now a mandatory study in nearly all the public schools of this country." It is estimated that there are now 16,000,000 children of school age in the United States under these Temperance Education laws. Mrs. Mary H. Hunt, of Boston, who edits the journal, is Superintendent for the World's and National Woman's Christian Temperance Union, and Life Director of the National Educational Association, writes the *Journal*, "We are reasonably sure this lesson, with its warning, will reach fully one-half million of these children;" a most gratifying assurance to every friend of humanity.

This is, indeed, striking at the root of the evil, and is the only way it can ever be eradicated. Alcohol,—whisky, is beyond doubt, the most baleful of all the evils with which mankind is cursed. It is the cause of more sin, suffering, sorrow, sickness, poverty, misery, *crime*, and death, and damnation, than all else combined. God speed these noble women in their righteous crusade.

